# Peripheral blood monocytes are responsible for γδ T cell activation induced by zoledronic acid through accumulation of IPP/DMAPP

**DOI:** 10.1111/j.1365-2141.2008.07435.x

**Published:** 2009-01

**Authors:** Anke J Roelofs, Marjo Jauhiainen, Hannu Mönkkönen, Michael J Rogers, Jukka Mönkkönen, Keith Thompson

**Affiliations:** 1 Bone and Musculoskeletal Research Programme, Institute of Medical Sciences, University of AberdeenAberdeen, UK; 2 Department of Pharmaceutics, University of KuopioKuopio, Finland

**Keywords:** bisphosphonates, alkylamines, monocytes, gamma delta T cells, isopentenyl diphosphate

## Abstract

Nitrogen-containing bisphosphonates indirectly activate Vγ9Vδ2 T cells through inhibition of farnesyl pyrophosphate synthase and intracellular accumulation of isopentenyl diphosphate (IPP) and dimethylallyl diphosphate (DMAPP), but the cells responsible for Vγ9Vδ2 T cell activation through IPP/DMAPP accumulation are unknown. Treatment of human peripheral blood mononuclear cells (PBMCs) with a pharmacologically relevant concentration of zoledronic acid induced accumulation of IPP/DMAPP selectively in monocytes, which correlated with efficient drug uptake by these cells. Furthermore, zoledronic acid-pulsed monocytes triggered activation of γδ T cells in a cell contact-dependent manner. These observations identify monocytes as the cell type directly affected by bisphosphonates responsible for Vγ9Vδ2 T cell activation.

Nitrogen-containing bisphosphonate drugs (N-BPs) are effective anti-resorptive agents commonly used for the treatment of postmenopausal osteoporosis and tumour-induced osteolysis. Intravenous administration of N-BPs is commonly associated with a flu-like acute-phase reaction involving the activation of Vγ9Vδ2 T cells (Kunzmann *et al*, 2000). As Vγ9Vδ2 T cells have potent anti-tumoural properties, activation and expansion of Vγ9Vδ2 T cells by N-BPs offers a promising strategy for cancer immunotherapy (Bonneville & Scotet, 2006). N-BPs, like dietary alkylamines, activate Vγ9Vδ2 T cells through inhibition of farnesyl diphosphate (FPP) synthase (Gober *et al*, 2003; Thompson & Rogers, 2004; Hewitt *et al*, 2005; Thompson *et al*, 2006a), most likely via an accumulation of the substrates of FPP synthase, isopentenyl diphosphate (IPP) and its stereoisomer dimethylallyl diphosphate (DMAPP), which are agonists of the Vγ9Vδ2 T cell receptor (TCR) (Tanaka *et al*, 1995). However, the exact cell type in peripheral blood that accumulates IPP/DMAPP after exposure to N-BP remains unknown. Monocytes have previously been shown to be required for activation of Vγ9Vδ2 T cells by the N-BP pamidronate (Miyagawa *et al*, 2001), although the role of monocytes was thought to be direct presentation of N-BP to the Vγ9Vδ2 TCR (Miyagawa *et al*, 2001). It has since become clear that the activation of Vγ9Vδ2 T cells by N-BPs involves intracellular N-BP uptake and IPP/DMAPP accumulation (Gober *et al*, 2003; Thompson & Rogers, 2004). We hypothesized that monocytes, due to their high endocytic activity, are the peripheral blood cells that efficiently internalize N-BP and accumulate IPP/DMAPP, and therefore are directly responsible for triggering Vγ9Vδ2 T cell activation.

## Materials and methods

### Reagents

Zoledronic acid (ZOL) and CGP-58318 (an analogue of ZOL; Legay *et al*, 2002) were provided by Novartis Pharma AG (Basel, Switzerland). All other reagents were from Sigma Chemical Company (Poole, Dorset, UK), unless otherwise stated. Mevastatin (MEV) was converted from the lactone form by dissolving 5 mg in 100 μl of ethanol and 100 μl 1 N NaOH, adding 1 ml phosphate buffered saline (PBS), and adjusting the pH to 8 using 1 N HCl. CGP-58318 was conjugated through the primary amine group to Alexa Fluor 488 (AF488-BP) or Alexa Fluor 680 (AF680-BP) as previously described for alendronate (Thompson *et al*, 2006b).

### *Cell culture and treatment*

This study was approved by the North of Scotland Research Ethics Committee. Informed consent was obtained for the collection of peripheral blood from healthy volunteers in accordance with the Declaration of Helsinki. Peripheral blood mononuclear cells (PBMCs) were isolated using Lymphoprep (Axis-Shield, Oslo, Norway) density gradient centrifugation and cultured in α-minimal essential medium supplemented with 100 U/ml penicillin, 100 μg/ml streptomycin, 2 mmol/l glutamine, 10% fetal bovine serum, and 10 U/ml recombinant human interleukin 2, except for detection of drug uptake where PBMCs were isolated using PharmLyse (BD Biosciences, Oxford, UK). Murine J774·2 macrophage-like cells were cultured as previously described (Thompson *et al*, 2006b), and plated at 5 × 10^5^ cells/well in 6-well plates and allowed to adhere overnight. The cells were then treated as indicated in the figure legends for 24 h before harvesting.

### *Detection and quantification of IPP/DMAPP and ApppI*

Human PBMCs (2 × 10^6^ cells/ml in 75 cm^2^ flasks) or J774·2 macrophages (5 × 10^5^ cells/well in 6-well plates) were treated as indicated in the figure legends, and T cells or monocytes were purified from human PBMCs by negative selection using MACS beads (Miltenyi Biotech GmbH, Bergisch Gladbach, Germany) according to manufacturer’s instructions. IPP/DMAPP and ApppI were quantified by high performance liquid chromatography negative ion electrospray ionization mass spectrometry (HPLC-ESI-MS) in acetonitrile extracts, as previously described (Mönkkönen *et al*, 2006), and results were expressed per milligram protein, determined by Bradford assay.

### *Detection of drug uptake*

**Fig 1:**
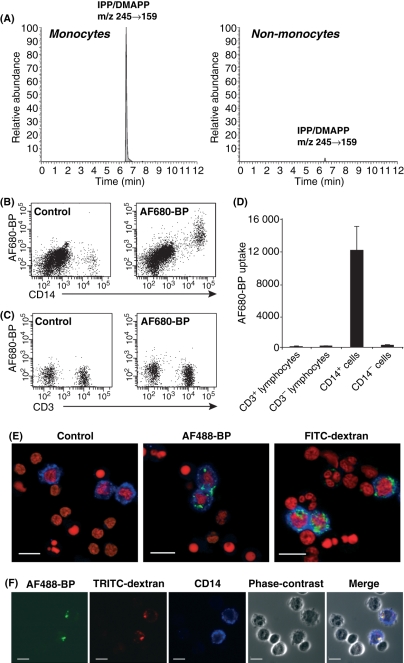
ZOL induces IPP/DMAPP accumulation selectively in monocytes and this is associated with high levels of intracellular drug uptake. (A) Human PBMCs were pulse-treated with 1 μmol/l ZOL for 2 h, washed and further cultured for 22 h in drug-free medium. Cells were purified into monocyte and non-monocyte fractions using magnetic bead separation and IPP/DMAPP was detected in acetonitrile cell extracts by HPLC-ESI-MS. Data shown are representative of two experiments from independent donors. Chromatograms were drawn on the same scale. No IPP/DMAPP was detected in extracts from untreated cells. (B–D) Human PBMCs were treated with 20 μmol/l AF680-BP for 24 h, washed, and stained with either anti-CD3-fluorescein isothiocyanate (FITC) or anti-CD14-FITC. Fixed cells were then analysed on an LSRII flow cytometer. Representative fluorescence scatter plots are shown of control cells and cells treated with AF680-BP, labelled with anti-CD14-FITC (B) or anti-CD3-FITC (C). CD3-stained cells were gated for the lymphocyte population based on forward *versus* side scatter profile. (D) Quantification of results shown in panels B and C using facsdiva software. Results are corrected for background fluorescence, and data are expressed as mean ± standard error of the mean relative fluorescence units (RFU) of nine independent donors from five experiments. (E–F) Human PBMCs were treated with (E) either 50 μmol/l AF488-BP (green) or 250 μg/ml FITC-dextran (green), or (F) 50 μmol/l AF488-BP (green) and 250 μg/ml TRITC-dextran (red), for 24 h. Cells were washed and stained with anti-CD14-allophycocyanin antibody (blue). Cell nuclei were visualised in (E) by counter-staining with SYTOX Orange (red). Formaldehyde-fixed and cytospun cells were analysed on a Zeiss LSM510 META system. White bars indicate 10 μm. Images show representative results from at least two independent donors.

Human PBMCs (1 × 10^6^ cells/well in 24-well plates) were treated as indicated in the legend of Fig 1, washed twice in ice-cold PBS, and stained with anti-CD14 (Miltenyi Biotech) or anti-CD3 antibodies (Beckman Coulter Immunotech, Palo Alto, CA, USA). Formaldehyde-fixed cells were analysed on an LSRII (BD Biosciences) flow cytometer using facsdiva software (BD Biosciences), or cytospun onto slides and counterstained with SYTOX Orange (Invitrogen, Paisley, UK) prior to analysis on a Zeiss LSM510 META system (Carl Zeiss Ltd, Welwyn Garden City, UK) using a 63× objective lens, and aim software (Carl Zeiss Ltd) for image acquisition and analysis.

### *Co-culture of ZOL-treated monocytes with γδ T cells*

Monocytes were isolated from human PBMCs using anti-CD14 magnetic bead separation (Miltenyi Biotec GmbH) according to the manufacturer’s instructions. Purity of the monocyte fraction was ≥93%, as assessed by CD14-labelling and flow cytometric analysis. Isolated monocytes were plated out at 5 × 10^5^ cells/well in 24-well plates, pulse-treated with 1 μmol/l ZOL or vehicle for 2 h, then washed with PBS. Vehicle- or ZOL-treated monocytes (1 × 10^5^ cells/well) were then co-cultured with the monocyte-depleted PBMCs (5 × 10^5^ cells/well) or with γδ T cells (1 × 10^4^ cells/well) isolated from the monocyte-depleted fraction using a negative γδ T cell isolation kit (Miltenyi Biotec GmbH), in a final volume of 500 μl medium in 24-well plates. To assess the role of cell–cell contact, transwell inserts (pore-size 0·4 μm) were used to separate monocyte-depleted PBMCs or γδ T cells from monocytes.

### *Detection of interferon-γ release*

After 72 h, conditioned medium was collected and the concentration of interferon (IFN)-γ was determined using an IFN-γ Quantikine enzyme-linked immunosorbent assay kit (R&D Systems, Minneapolis, MN, USA) according to the manufacturer’s instructions.

### *Detection of γδ T cell proliferation*

After 7 d, the percentage of Vδ2-TCR^+^ T cells in the PBMC co-cultures was determined by immunostaining with anti-CD3 and anti-Vδ2-TCR antibodies and flow cytometric analysis, as previously described (Thompson *et al*, 2006a).

## Results and discussion

### ZOL and sec-butylamine induce IPP/DMAPP accumulation in J774·2 macrophages and this is prevented by mevastatin

Recent studies have suggested that accumulation of IPP/DMAPP triggers Vγ9Vδ2 T cell activation by N-BPs as well as alkylamines (Gober *et al*, 2003; Thompson & Rogers, 2004; Hewitt *et al*, 2005; Thompson *et al*, 2006a). However, the IPP/DMAPP accumulation in response to pharmacological inhibition of FPP synthase is not well characterized. We used quantitative HPLC-ESI-MS to assess intracellular IPP/DMAPP levels in response to ZOL treatment in J774·2 macrophages. Low concentrations of ZOL (≥0·5 μmol/l) were sufficient to cause detectable accumulation of IPP/DMAPP (Fig S1A). In addition, the alkylamine sec-butylamine (SBA) also induced IPP/DMAPP accumulation at concentrations ≥5 mmol/l (Fig S1A). The relatively high concentration of SBA required to induce IPP/DMAPP accumulation correlated with the far lower potency of alkylamines for inhibiting FPP synthase than ZOL (Thompson *et al*, 2006a). Simultaneous treatment with MEV almost completely prevented the ZOL- or SBA-induced IPP/DMAPP accumulation (Fig S1A). These findings provide further evidence for the notion that statins, through inhibiting HMG-CoA reductase upstream of FPP synthase, prevent N-BP- and alkylamine-induced Vγ9Vδ2 T cell activation by preventing the accumulation of IPP/DMAPP resulting from FPP synthase inhibition (Gober *et al*, 2003; Thompson & Rogers, 2004; Hewitt *et al*, 2005; Thompson *et al*, 2006a), and further confirm the mechanism of Vγ9Vδ2 T cell activation and proliferation by FPP synthase inhibitors. The accumulation of IPP/DMAPP in J774·2 cells in response to both ZOL and SBA was associated with a concurrent accumulation of the recently identified novel IPP metabolite ApppI (Mönkkönen *et al*, 2006), and this was also blocked by MEV (Fig S1B). Whether the metabolism of IPP into ApppI affects the activation of Vγ9Vδ2 T cells by N-BPs or alkylamines is presently unknown.

### *Monocytes accumulate IPP/DMAPP in response to ZOL in human PBMC cultures*

To investigate which cell type in peripheral blood is responsible for the IPP/DMAPP accumulation and resulting Vγ9Vδ2 T cell activation by N-BPs, we assessed intracellular IPP/DMAPP levels in human PBMC cultures in response to ZOL treatment. IPP/DMAPP could not be detected in untreated cells, but when human PBMCs were pulsed for 2 h with 10 μmol/l ZOL, IPP/DMAPP was clearly detectable in the non-T cell fraction, whereas T cells showed only minor levels of IPP/DMAPP (Fig S2). The MS/MS spectrum from the peak in the non-T cell fraction was identical to the MS/MS spectrum of synthetic IPP (not shown). When human PBMCs were pulsed with 1 μmol/l ZOL for 2 h (to mimic the estimated circulating concentration and exposure of peripheral blood cells following a standard dose of ZOL *in vivo*; Chen *et al*, 2002), ZOL-induced IPP/DMAPP accumulation was clearly detectable in monocytes purified by magnetic bead separation, but hardly detectable in the non-monocyte fraction (Fig 1A). Isolation of monocytes based on cell adherence yielded similar results (not shown).

### *Peripheral blood monocytes efficiently internalize N-BP*

We have shown recently that fluid-phase endocytosis is the major route by which N-BPs are internalized in J774·2 macrophages and osteoclasts (Thompson *et al*, 2006b). Since peripheral blood monocytes are highly endocytic, we investigated whether the selective accumulation of IPP/DMAPP in this cell type correlated with relatively high levels of drug uptake. Indeed, in cultures of PBMCs, only CD14^+^ monocytes internalized large amounts of fluorescently labelled N-BP, while little or no uptake by other cell types, including CD3^+^ lymphocytes, could be detected at the concentrations and treatment duration used (Fig 1B-D). Similarly, only monocytes internalized large amounts of dextran (Fig 1E), a marker of fluid-phase endocytosis, and confocal microscopy demonstrated intracellular co-localization of fluorescently labelled N-BP with dextran (Fig 1F). This suggests that N-BP uptake by peripheral blood monocytes, similar to that by J774·2 macrophages and osteoclasts (Thompson *et al*, 2006b), predominantly occurs via fluid-phase endocytosis. Together, these findings indicate that, following an intravenous infusion of ZOL, only highly endocytic monocytes in peripheral blood are likely to internalize sufficient ZOL to cause a significant accumulation of IPP/DMAPP.

### *ZOL-pretreated monocytes activate Vγ9Vδ2 T cells in human PBMC cultures, and this is dependent on cell–cell contact*

**Fig 2:**
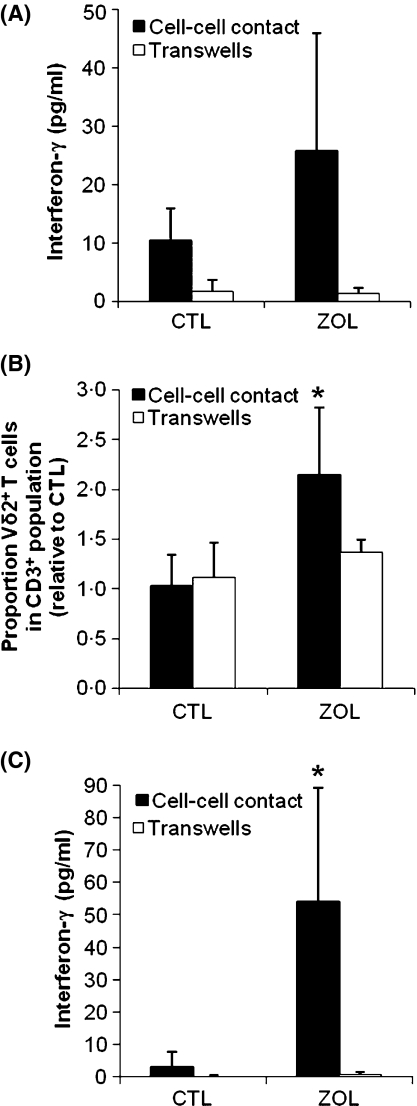
Activation of Vγ9Vδ2 T cells by ZOL-treated monocytes. Monocytes were purified from human PBMCs by CD14 magnetic bead isolation and pulsed with vehicle or 1 μmol/l ZOL for 2 h. Vehicle- or ZOL-treated monocytes were then co-cultured with monocyte-depleted PBMCs (A, B) or γδ T cells obtained from monocyte-depleted PBMCs using a γδ T cell magnetic bead isolation kit (C). Transwell inserts were used to separate the monocytes from the PBMCs or the enriched γδ T cells. After 72 h, the concentration of IFN-γ in the cell culture medium was determined by enzyme-linked immunosorbent assay (A, C). To determine proliferative responses, the proportion of Vδ2^+^ T cells in the CD3^+^ population was determined by immunolabelling and flow cytometric analysis after 7 d of culture and expressed relative to control (B). Data is shown as mean ± standard deviation of 3 independent donors. *P< 0·05 as compared to vehicle-treated control.

Having shown that monocytes accumulate detectable levels of IPP/DMAPP following treatment with a clinically relevant pulse of ZOL (1 μmol/l for 2 h), we investigated whether purified peripheral blood monocytes pretreated with the same concentration and duration of ZOL activated Vγ9Vδ2 T cells in co-cultures with untreated (monocyte-depleted) PBMCs. ZOL-treated monocytes induced a 2·5 ± 1·2-fold (*n* = 3) increase in IFN-γ release when co-cultured with monocyte-depleted PBMCs, as compared to co-culture of monocyte-depleted PBMCs with vehicle-treated monocytes (Fig 2A), although this did not reach statistical significance. The increase in IFN-γ correlated with a 2·1 ± 0·5-fold (*n* = 3) increase (*P* < 0·05) in Vδ2^+^ T cell number after a 7 d culture (Fig 2B), and an increase in cell clustering (not shown) in co-cultures with ZOL-treated monocytes as compared to co-cultures with vehicle treated-monocytes. Both IFN-γ release and Vδ2^+^ T cell proliferation were prevented when the monocyte-depleted PBMCs were separated from monocytes using transwell inserts, indicating that cell–cell contact is required for the activation of Vγ9Vδ2 T cells by ZOL-treated monocytes.

### *ZOL-pretreated monocytes activate purified γδ T cells*

Finally, we investigated whether ZOL-pretreated monocytes activated Vγ9Vδ2 T cells in enriched γδ T cell cultures. After 72 h, IFN-γ release was significantly increased in co-cultures of γδ T cells with ZOL-treated monocytes, compared to co-cultures with vehicle-treated monocytes (*P* < 0·05; Fig 2C). As observed in the co-cultures of monocytes with monocyte-depleted PBMCs, the increase in IFN-γ release was abrogated when γδ T cells were separated from monocytes using transwell inserts (Fig 2C). This strongly suggests that ZOL-treated monocytes can directly activate γδ T cells via a cell contact-dependent mechanism.

## Conclusions

This study demonstrated that ZOL induced IPP/DMAPP accumulation selectively in monocytes following treatment with a clinically-relevant dose and duration of ZOL, most probably a result of efficient intracellular drug uptake by this highly endocytic cell type. Furthermore, peripheral blood monocytes pretreated with ZOL induced activation and proliferation of Vγ9Vδ2 T cells, both in mixed PBMC cultures, and in co-cultures with enriched γδ T cells. These findings provide a novel explanation for why monocytes are essential for Vγ9Vδ2 T cell activation by N-BPs, and suggest that monocytes, following exposure to ZOL, can directly activate Vγ9Vδ2 T cells in a cell contact-dependent manner.

## Supporting information

**Figure S1.** Mevastatin prevents both ZOL- and SBA-induced IPP/DMAPP and ApppI accumulation in J774.2 macrophages.

**Figure S2.** ZOL-induced IPP/DMAPP accumulation occurs predominantly in non-T cells in human PBMC cultures.
