# Supplementary figures and images for: Peripheral blood monocytes are responsible for γδ T cell activation induced by zoledronic acid through accumulation of IPP/DMAPP
Source: Br J Haematol. 2009 Jan;144(2):245–50. doi: 10.1111/j.1365-2141.2008.07435.x (PMC2659391; doi:10.1111/j.1365-2141.2008.07435.x)

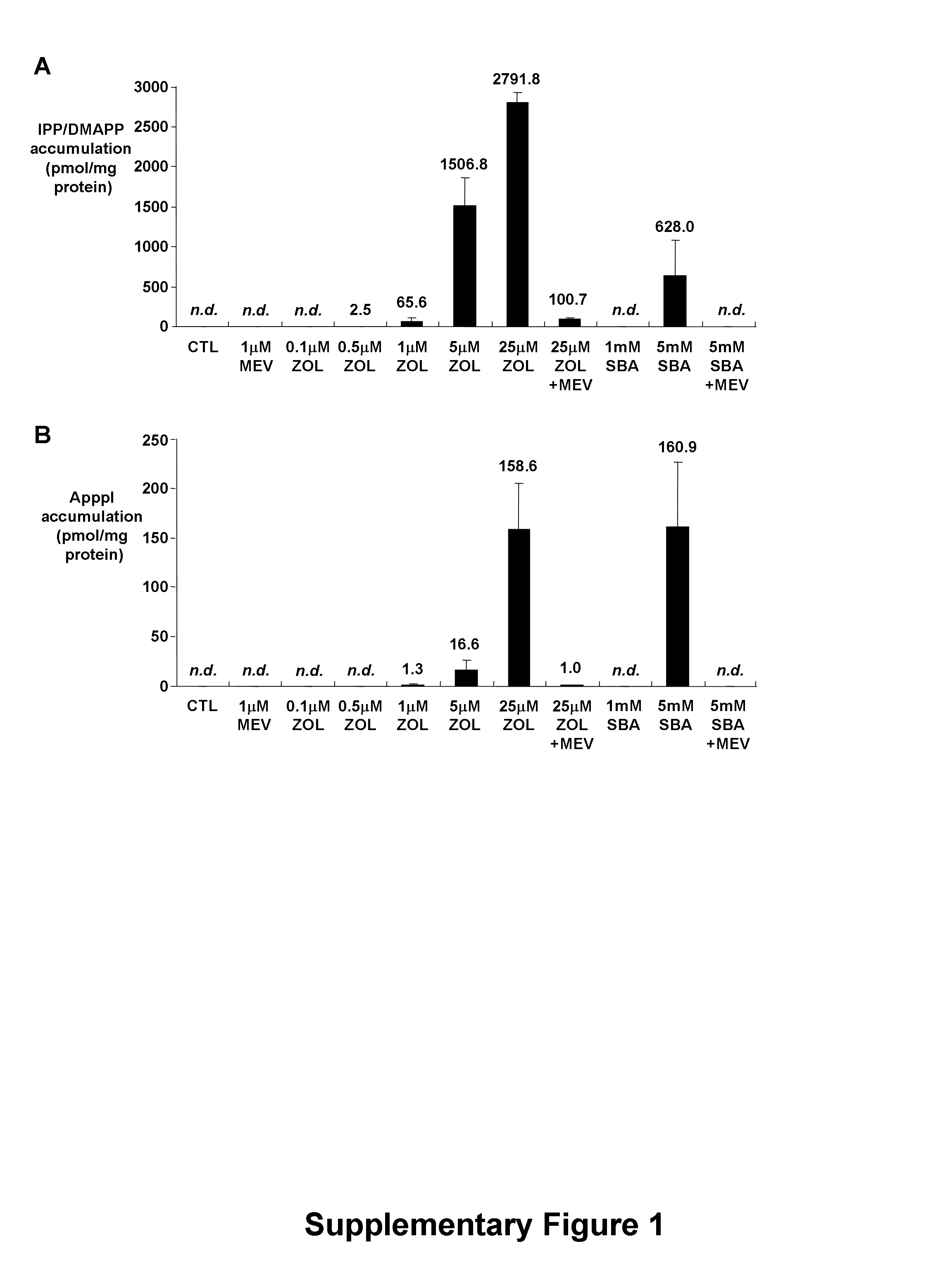

Supplement: Supplementary file 1 [file bjh0144-0245-SD1.tif]

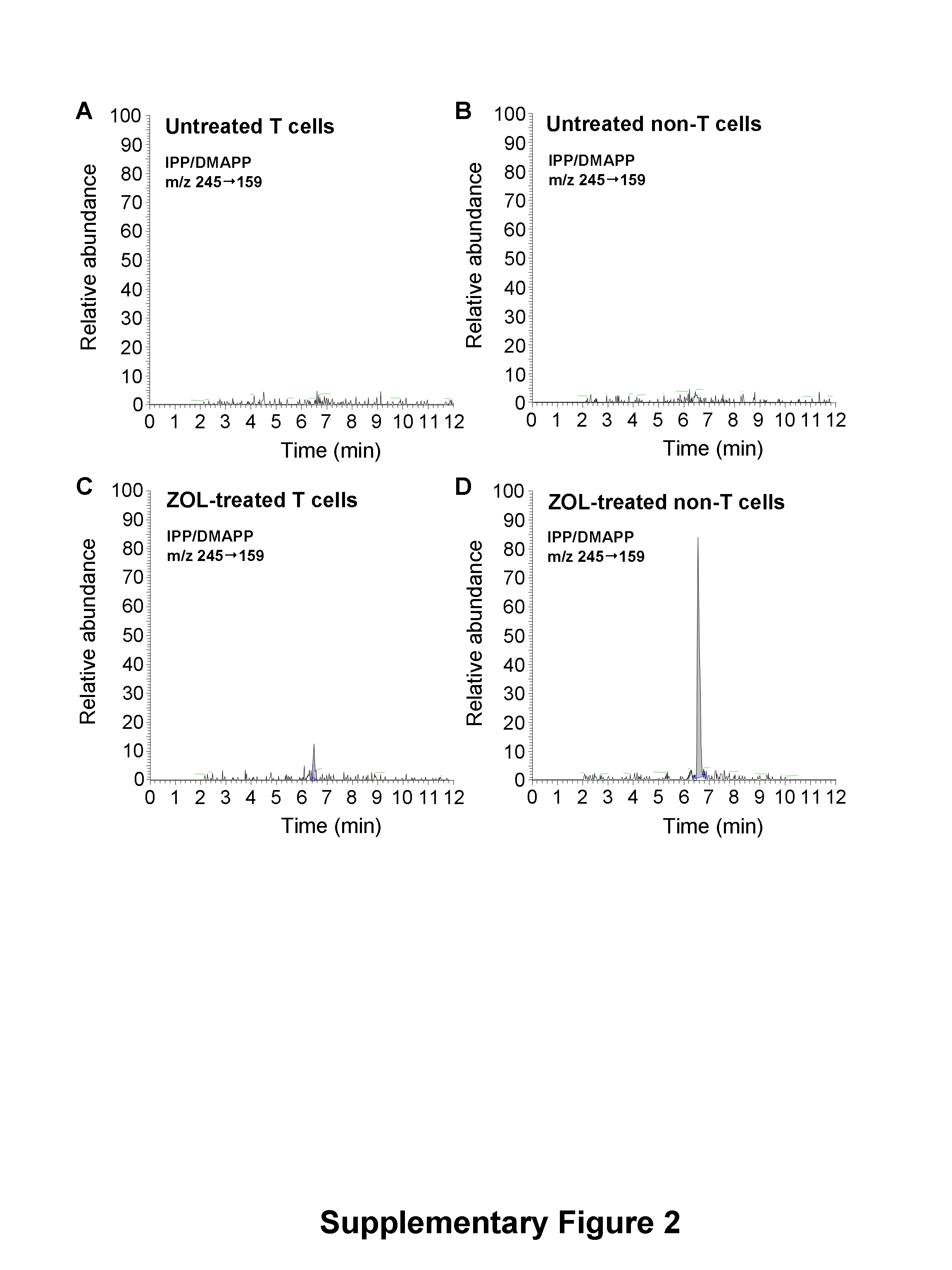

Supplement: Supplementary file 2 [file bjh0144-0245-SD2.tif]
