# Supplementary material for: Peripheral blood monocytes are responsible for γδ T cell activation induced by zoledronic acid through accumulation of IPP/DMAPP
Source: Br J Haematol. 2009 Jan;144(2):245–50. doi: 10.1111/j.1365-2141.2008.07435.x (PMC2659391; doi:10.1111/j.1365-2141.2008.07435.x)
Supplement: Supplementary file 3 [file bjh0144-0245-SD3.doc]

**Supplementary Fig 1.** Mevastatin prevents both ZOL- and SBA-induced IPP/DMAPP and ApppI accumulation in J774.2 macrophages. J774.2 macrophages were treated with 1 μM MEV, 0.1–25 μM ZOL, 25 μM ZOL + 1 μM MEV, 1-5 mM SBA, 5 mM SBA + 1 μM MEV, or 1 mM 3-PEHPC, for 24 h. Cells were harvested, washed in PBS and lysed in acetonitrile before levels of IPP/DMAPP (A), or ApppI (B) were quantified using HPLC-ESI-MS. Data shown are the mean + S.E.M. of at least three independent experiments. *n.d.*: not detected (IPP detection limit 2.5 pmol/mg; ApppI detection limit 1 pmol/mg).

**Supplementary Fig 2.** ZOL-induced IPP/DMAPP accumulation occurs predominantly in non-T cells in human PBMC cultures. Human PBMCs were pulse-treated with 10 μM ZOL for 2 h, washed and further cultured for 22 h in drug-free medium. Cells were purified into T cell and non-T cell fractions using a T cell negative isolation kit (Miltenyi Biotec) and IPP/DMAPP was detected in acetonitrile cell extracts by HPLC-ESI-MS. (A) untreated T cell fraction; (B) untreated non-T cell fraction; (C) ZOL-treated T cell fraction; (D) ZOL-treated non-T cell fraction. Data shown are representative of two experiments from independent donors. Chromatograms were drawn on the same scale.
